# A Systematic Review on the Effects of Botanicals on Skeletal Muscle Health in Order to Prevent Sarcopenia

**DOI:** 10.1155/2016/5970367

**Published:** 2016-03-09

**Authors:** M. Rondanelli, A. Miccono, G. Peroni, F. Guerriero, P. Morazzoni, A. Riva, D. Guido, S. Perna

**Affiliations:** ^1^Department of Public Health, Experimental and Forensic Medicine, Section of Human Nutrition, Endocrinology and Nutrition Unit, Azienda di Servizi alla Persona, University of Pavia, 27100 Pavia, Italy; ^2^Department of Clinical Sciences, Faculty of Medicine and Surgery, University of Milan, Milan, Italy; ^3^Azienda di Servizi alla Persona, Pavia, Italy; ^4^Research and Development Unit, Indena, 20139 Milan, Italy; ^5^Department of Brain and Behavioral Sciences, Medical and Genomic Statistics Unit, University of Pavia, 27100 Pavia, Italy; ^6^Department of Public Health, Experimental and Forensic Medicine, Biostatistics and Clinical Epidemiology Unit, University of Pavia, 27100 Pavia, Italy

## Abstract

We performed a systematic review to evaluate the evidence-based medicine regarding the main botanical extracts and their nutraceutical compounds correlated to skeletal muscle health in order to identify novel strategies that effectively attenuate skeletal muscle loss and enhance muscle function and to improve the quality of life of older subjects. This review contains all eligible studies from 2010 to 2015 and included 57 publications. We focused our attention on effects of botanical extracts on growth and health of muscle and divided these effects into five categories: anti-inflammation, muscle damage prevention, antifatigue, muscle atrophy prevention, and muscle regeneration and differentiation.

## 1. Introduction

Sarcopenia is the loss of muscle protein mass and of muscle function and it occurs with increasing age, being a major component in the development of frailty [1]. It is a syndrome characterized by the progressive and generalized loss of skeletal muscle mass and strength with a risk of adverse outcomes such as physical disability, poor quality of life, and death [2]. Preventative diet, exercise, or treatment interventions particularly in middle-aged adults at the low end of the spectrum of muscle function may help to preserve mobility in later years and improve health span [3]. The therapeutic options for sarcopenia are unclear and constantly evolving. The most rational approach to delay the progression of sarcopenia is based on the combination of proper nutrition, possibly associated with the use of dietary supplements, and a regular exercise program [4]. Despite the major advantages offered by natural therapies with their long traditional use and poor physiological and psychological addiction as is commonly seen with conventional medicine [5], few studies have been performed on the topic of age-correlated pathologies of skeletal muscle. The aim of this review was to investigate the effectiveness of botanicals on skeletal muscle health focusing on possible therapeutics approaches to prevent sarcopenia.

## 2. Materials and Methods

The present systematic review was performed according to the steps by Egger et al. [6] (Table 1), as follows: (i) configuration of a working group: three operators skilled in clinical nutrition in the geriatric age, of whom one was acting as a methodological operator and two were participating as clinical operators; (ii) formulation of the revision question on the basis of considerations made in the abstract: “sarcopenia and muscle mass, use of botanic extracts during aging”; (iii) identification of relevant studies: a research strategy was planned, on PubMed and Scopus, as follows: (a) definition of the key words (sarcopenia, muscle mass, inflammation, antioxidants, botanical extracts, phytotherapy, muscle atrophy, muscle fatigue,* Camellia sinensis*,* Vitis vinifera*,* Zingiber officinale*,* Citrus aurantium*, and* Panax quinquefolius*), allowing the definition of the interest field of the documents to be searched, grouped in inverted commas (“…”), and used separately or in combination; (b) use of the Boolean AND operator, which allows the establishment of logical relationships among concepts; (c) research modalities: advanced search; (d) limits: papers published until June 2015; humans, animals, in vivo, and in vitro studies; languages: English; (e) manual search performed by the senior researchers, experienced in clinical nutrition, through the revision of reviews and research articles on sarcopenia in the elderly, published in qualified journals of the Index Medicus.

**Figure 1:**
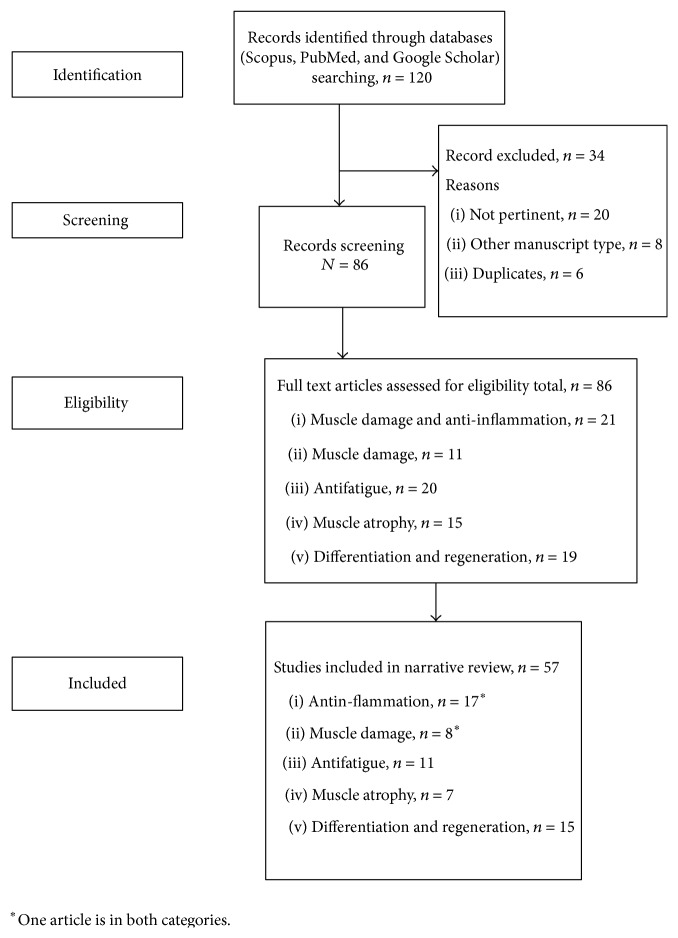
Flow diagram of narrative review of the literature.

Analysis and presentation of the outcomes had been done as follows: the data extrapolated from the revised studies were summarized in Tables 2–6; in particular, for each study, we specified the author and year of publication, the plant and the active principles, the models used, the posology, and the main results obtained. The analyses were carried out in the form of a narrative review of the reports. The flow diagram of narrative review of the literature has been reported in Figure 1. As shown in Figure 1, we consider several effects of botanical extracts on growth and health of muscle; we divided these effects into five categories: anti-inflammatory activity, muscle damage prevention, antifatigue, muscle atrophy prevention, and muscle regeneration and differentiation. After this, we examined the typology of the studies, that is, in vitro, animals (mice, rats), or human, and we classified the human data according to the different condition such as postmenopausal women, athletes or others. Another key point was to identify the dosage of the extracts for each trial and the botanical compounds responsible for the activity. The use of the databases as PubMed or Scopus was determinant to enrich our review. At the end, we reported an analysis of all plants and their extracts that have a beneficial role in preventing sarcopenia or improve muscle health condition.

## 3. Results

### 3.1. Screening and Selection Process of Study

Of 120 articles identified, 57 studies met inclusion criteria (Figure 1), including 17 focused on anti-inflammation, 8 focused on muscle damage, 11 based on antifatigue effects, 7 based on muscle athorpy, and 15 based on muscle differentiation and regeneration (Figure 1).

As reported in Table 2, there are different effects on skeletal muscle for each botanical. At present, we evaluated in the literature over 70 different mechanisms of action.

### 3.2. Anti-Inflammatory Activity

This research has been carried out based on the keywords “skeletal muscle mass” and “inflammation” and “botanicals” or “plants” or “extracts”; 21 articles were sourced and 17 studies are taken into account. Among these papers, 3 studies are in in vitro setting, 4 in animals, 8 in humans, and two both in animals and in in vitro setting (Table 3).

Inflammation and oxidative stress induce muscle damage and muscle pain [18] and several botanicals (*Phlebodium decamanum*,* Citrus aurantium*,* Coffea arabica*,* Zingiber officinale*,* Eugenia punicifolia*,* Panax ginseng*,* Go-sha-jinki-Gan*,* Vitis vinifera*, and* Curcuma longa* L.) have a significant role in the prevention of this phenomenon.

Supplementation with* Phlebodium decamanum* (5 capsules of 400 mg) reduces inflammatory response and also the degree of oxidative stress in human during high-intensity exercise, through the decrease of 8-hydroxy-2′-deoxyguanosine and isoprostanes generation, the increase of antioxidant enzyme activities in erythrocyte and total antioxidant status in plasma, the decrease of tumor necrosis factor (TNF-*α*), and the increase of soluble receptor II of TNF-*α* (sTNF-RII), but kept the levels of interleukin-6 (IL-6) and interleukin-1 antagonist receptor (IL-1ra) [18]. Other studies examine the anti-inflammatory effect of flavonoids isolated from* Citrus aurantium*,* Coffea arabica*, and* Zingiber officinale* on interleukins such as IL-1*α* and IL-6 and TNF-*α* on skeletal muscle cells. Specifically, the flavonoids (hesperidin, nobiletin, and naringin of* Citrus aurantium*, also known as sour orange) inhibit the inflammatory response in lipopolysaccharide- (LPS-) induced L6 skeletal muscle cells. In addition, the flavonoids isolated from Korean* Citrus aurantium* L. inhibit significantly inducible nitric oxide synthase (iNOS), cyclooxygenase-2 (COX-2), IL-6, and TNF-*α* by blocking the nuclear factor-kappa B (NF-*κ*b) and by blocking mitogen-activated protein kinases (MAPKs) signal pathways. Another study in the same muscle cells demonstrates the anti-inflammatory role of flavonoids isolated from* Citrus aurantium* through the modulation in protein related to the immune response. Furthermore, the pretreatment with flavonoids resulted in a decreased level of cleaved caspase-3, which is induced by muscle inflammation and is involved in muscle proteolysis and atrophy. Also,* Zingiber officinale*, commonly known as ginger, showed interesting anti-inflammatory and analgesic effects in humans who ingested 2 grams of ginger or placebo after exercise; however, this extract has no remarkable effect after single administration. In fact, only a moderate reduction in the progression of muscle pain from 24 h to 48 h following eccentric exercise was observed in participants who consumed ginger 24 h after exercise, and this effect was not enhanced by heat-treated ginger. In mice,* Coffea* decreases the levels of interleukins IL-1*α* and IL-6 and TNF-*α*, which are correlated with muscle weight and grip strength. Using mice cells in vitro, coffee increases the number of proliferating cells and augmented DNA synthesis through the Akt signaling pathway. As a result, there is a combination of augmented satellite cell activation and decreased inflammatory levels by coffee treatment; it has anti-inflammatory effects both because it has antioxidant properties and because it has compounds, such as kahweol, with immunomodulatory properties [7, 19, 24, 54, 61]. Also,* Eugenia punicifolia* showed anti-inflammatory properties in the gastrocnemius muscle of mdx dystrophic mice; in particular, the activity of dichloromethane fraction of* Eugenia punicifolia* (Ep-CM), in mice, decreases metalloprotease-9 and metalloprotease-2 activities (indicators of local inflammation and tissue remodeling, resp.) and levels of tumor necrosis factor-*α* and NF-*κ*B transcription factor [8]; isolated pentacyclic triterpene from* Eugenia punicifolia* reduces myoblast cells proliferation, has no effects on apoptosis, and increases matrix metalloproteases and muscular area (MMP-9 and MMP-2) [12]. As shown in the study by Yu et al. [10], Dammarane steroids (DS) of* Panax ginseng* produce anti-inflammatory effects in rats, following muscle damage exercise, because they potentiate inflammation at baseline but exerted anti-inflammatory effects on skeletal muscle following muscle-damaging exercise. Another study has also highlighted the effect of steroid Rg1 (capsule with 5 mg of Rg1), an ergogenic component of ginseng, in healthy human against exercise challenge: the extract can minimize unwanted lipid peroxidation and attenuate proinflammatory shift under exercise challenge and so it ameliorates the postexercise recovery and mitochondria enzyme adaptation probably because the incorporation of the bulky steroid moiety of Rg1 into cellular membrane lipid may enhance molecular complexity and mechanical stability of the cell and mitochondrial membranes [17].* Panax notoginseng*, as shown by Pumpa et al., seems to have no particular effects on interleukins, indicators of inflammation and muscle damage, in well-trained males after a bout of eccentric exercise designed to induce delayed-onset muscle soreness (DOMS) (in the experiment, 400 mg of* Panax notoginseng* was used) [55]. Even* Go-sha-jinki-Gan* (GJG) maintains the area of muscle fibers in the soleus via normalizing signal transduction through the insulin-growth factor (IGF-1) Akt axis, the suppression of inflammation, and the maintenance of mitochondrial-related transcription factors in mice [28]. A positive effect on cell atrophy caused by TNF-*α* was shown with resveratrol (in* Vitis vinifera*) supplementation in a muscle cell line (regulating the Akt/mTOR/FoxO1 signaling pathways together with inhibition of the atrophy-related ubiquitin ligase) [16].

Finally, several studies have investigated the mechanisms by which curcumin, a constituent of turmeric (*Curcuma longa* L.), exerts its beneficial effect on muscle [62]. Early experimental study demonstrated that curcumin suppresses the activation of NF-*κ*B, an effect of critical relevance in DOMS relief, since NF-*κ*B appears to be involved in the regulation of proteolysis and inflammation in muscle [62]. Therefore, inhibition of NF-*κ*B by curcumin may result in a muscle-protective effect. Consistently, it has been suggested that curcumin may prevent loss of muscle mass during sepsis and endotoxaemia and may stimulate muscle regeneration after traumatic injury [62]. Other mechanisms potentially responsible for the anti-inflammatory and antioxidant properties of curcumin include induction of heat-shock response [62], reduction in the expression of the proinflammatory enzyme cyclooxygenase-2 (COX-2), and promotion of the antioxidant response by activation of the transcription factor Nrf2 [63]. More recent studies confirm that curcumin can reduce inflammation and decrease some of the negative effects associated with eccentric exercise-induced muscle damage, including the release of proinflammatory cytokines and markers of muscle injury like creatine kinase (CK), as shown in animal models [31] and in in vitro settings [64].

The three studies that have been conducted until now in humans [22, 23, 30] have shown that curcumin, at the dosages of 1 g twice daily (as the Phytosome® delivery system, Meriva®) and 2.5 g twice daily, and 150 mg of solid-lipid nanoparticle curcumin (Theracurmin®), respectively, can prevent DOMS with some evidence of enhanced recovery of muscle performance, maximal voluntary contraction loss, and serum creatine kinase activity increase.

In conclusion, the muscle that makes activities undergoes an increase in inflammation that can damage the muscle itself. It is important to counteract the inflammatory activity in order to preserve the muscle from numerous types of damage. Several animal and in vitro studies have investigated the efficacy of botanicals with recognized anti-inflammatory activity (such as* Phlebodium decamanum*,* Citrus aurantium*,* Coffea arabica*,* Zingiber officinale*,* Eugenia punicifolia*,* Panax ginseng*,* Go-sha-jinki-Gan*,* Vitis vinifera*, and* Curcuma longa* L.) on inflammation secondary to muscle activity (Table 2). These botanical extracts exerted their effects through different biochemical pathways, specifically decreasing interleukins or aging on transcriptional factors. Human studies were performed using four botanicals (*Panax ginseng*,* Zingiber officinale*,* Phlebodium decumanum*, and* Curcuma longa* L.) showing that (1) the daily consumption of raw and heat-treated* Zingiber* resulted in moderate-to-large reductions in muscle pain after exercise-induced muscle injury; (2)* Phlebodium* supplementation for both professional and amateur athletes performing strenuous exercise resulted in reducing the undesirable effects of the oxidative stress and inflammation signaling elicited during high-intensity exercise; (3)* Panax notoginseng* did not convincingly have an effect on performance, muscular pain, or assessed blood markers in well-trained males after an intense bout of eccentric exercise that induced delayed-onset muscle soreness (DOMS); (4) curcumin could prevent DOMS enhancing the recovery of muscle performance and the maximal voluntary contraction loss and modulating the serum creatine kinase activity increase.

All these clinical studies considered the reduction of inflammation and consequently muscle pain after a strenuous exercise and not in sarcopenic subjects, but this is a good starting point for the future utilization of these plants in the elderly.

### 3.3. Muscle Damage Prevention

This research has been carried out based on the keywords “skeletal muscle mass” and “damage” and “botanicals” or “plants” or “extracts”; 11 articles were sourced and 8 studies have been taken into consideration. Among these, 2 studies are in in vitro setting, 3 in animals, and 3 in humans (Table 4).

A recent study by Kawanishi et al. has clarified properties of* curcumin* after downhill running-induced muscle damage in mice. This study underlines how curcumin has an antioxidant effect in mice following downhill running-induced muscle damage; however, no differences in plasma creatine kinase (CK) and plasma lactate dehydrogenase (LDH), as markers of muscle damage, were observed. Curcumin administration immediately after downhill running did not prevent muscle damage but significantly attenuates the concentration of hydrogen peroxide and NADPH-oxidase gene expression; therefore, curcumin may be beneficial for the prevention of oxidative stress in downhill running-induced skeletal muscle damage [49]. Two recent studies in humans by Pumpa et al. [34] and Matsumura et al. [55] investigated the effects of* Panax notoginseng* (4000 mg) and* Zingiber officinale* (4 g for 5 days) on delayed-onset muscle soreness (DOMS);* Zingiber officinale* supplementation could have accelerated the recovery of maximal strength following muscle damage but did not prevent delayed muscle damage. The authors concluded that there is no evidence to support the use of* Panax* as a preventive option for DOMS and its related inflammation.* Rhodiola rosea* (600 mg/d) did not attenuate the postmarathon decrease in muscle function, the increases in muscle damage, the extracellular heatshock protein (eHSP72), or the plasma cytokines in human experienced runners [56]; however, the same plant modulates in vitro the expression of molecular factors (chaperone HSP70) such as heatshock proteins (HSP) in order to protect C2 C12 myotubes cells against peroxide-induced oxidative stress, suggesting a potential antioxidant role [44]. Finally, Haramizu et al. demonstrated that catechins of* Camellia sinensis* attenuate downhill running-induced muscle damage in mice, perhaps through their antioxidant properties, hastening recovery of physical performance [20]. A typical example of muscle damage is the cellular dysfunction caused by lipid excess. Lipid excess activates endoplasmatic reticulum (ER) stress in skeletal muscle and, as a consequence, accumulation of unfolded or misfolded proteins in ER lumen. Rodriguez et al. demonstrated that epigallocatechin-3-gallate (EGCG) from* Camellia sinensis* could protect mice muscle against ER stress, especially thanks to its antioxidant properties [50]. Dargelos et al. investigate the role of a natural antioxidant extracted from pine bark (*Pinus pinaster*) in cultured human skeletal muscle satellite cells. Results showed that this polyphenolic extract is able to protect cells from oxidative stress (H_2_O_2_) damage and prevent the apoptosis and the activation of calpains mediated by H_2_O_2_ [46].

In conclusion (Table 3), until today, important studies were made on humans and animals for the prevention of muscle damage. Most of the plants used (*Curcuma longa*,* Panax notoginseng*,* Zingiber officinale*,* Rhodiola rosea*,* Camellia sinensis*, and* Pinus pinaster*) act on DOMS, thanks to their antioxidant properties. In human,* Panax notoginseng* seems to have no effect as a preventive option for DOMS, and* Rhodiola rosea* does not attenuate muscle damage. Further studies are needed but we can say that botanical supplementation, thanks to its antioxidant properties, could be useful to prevent sarcopenia due to the fact that the loss of muscle mass in aging is driven also by oxidative stress, as it happens after strenuous exercise.

### 3.4. Antifatigue

This research has been carried out based on the keywords “skeletal muscle mass” and “fatigue” and “botanicals” or “plants” or “extracts”; 20 articles were sourced and 11 studies are taken into account. Among these, only one study is made in humans, one in in vitro settings, and one both in in vitro settings and in animals and the others are made in animals (Table 5).

Tan et al. in 2013 investigated for the first time the role of ginsenoside Rb1 (Grb1) in* Panax quinquefolius*, as antifatigue agent, on postoperative fatigue syndrome (POFS) in a rat model induced by major small intestinal resection, through its antioxidant properties and the improvement of energy metabolism. Grb1 enhances maximum grip strength and increases the activity of lactate dehydrogenase and other biochemical parameters. The results suggested that GRb1 improves the maintenance of normal pH range in muscle tissue by reducing the accumulation of lactic acid (LA) and attenuates LA induced side effects of various biochemical and physiological processes, which impair bodily performance [41]. In accordance, the study by Nallamuthu et al. demonstrated the antifatigue properties in mice of* A*.* marmelos* fruit, most probably manifested by delaying the accumulation of serum lactic acid, increasing the fat utilization, and upregulating the skeletal muscle metabolic regulators [39]. Likewise,* Salvia sativa*,* Angelica sinensis*,* Cucurbita moschata*,* Withania somnifera*, and* Acanthopanax senticosus* extracts exhibit different antifatigue effects. All of these studies, with the exception of* Withania somnifera*, are performed in animals (rats or mice). These studies demonstrate that the antioxidant properties of plants play an important role in reducing fatigue.* Salvia* reduces lipid peroxidation, lactate dehydrogenase, creatine kinase activities, enhanced antioxidant enzymes, and cholinesterase (ChE) activities in the skeletal muscle of endurance exercise rats; similar effects have been observed for other extracts, with some differences between each other, in which, additionally, antifatigue is measured also by forelimb grip strength and exhaustive swimming time as well as serum levels of lactate, ammonia, glucose, and creatine kinase after a 15 min swimming exercise. Specifically, the mechanisms of* Acanthopanax* (also called* Eleutherococcus senticosus* or Siberian ginseng) are the reduction of the level of triglycerides by increasing fat utilization, the delay of the accumulation of blood urea nitrogen (BUN), and the increase of the lactate dehydrogenase (LDH) to reduce the accumulation of lactic acid in muscle and then protect the muscle tissue [32, 33, 36, 37, 42]. Strange but active is Tao-Hong-Si-Wu-Tang that shows antifatigue activity in mice due to extended exhaustive swimming time, the increase of liver and muscle glycogen contents, and the decrease of the lactic acid (BLA) and urea nitrogen (BUN) plasmatic contents [40]. Also, Chen et al. define the antifatigue property of* Rubus parvifolius* L. (RPL) in experiment with mice, finding that total saponins from RPL possess potent capabilities to alleviate fatigue induced by forced swimming and that nigaichigoside F1 was responsible for the pharmacological effect. The underlying mechanisms include delays in the accumulation of serum urea nitrogen (SUN) and lactic acid (LA), a decrease in TG level by increasing fat consumption, increases in hepatic glycogen (HG) and LDH so that lactic acid accumulation was decreased, the reduction of ammonia in the muscle, and the suppression of increased immune activation and inflammatory cytokine production [21].* Viscum album* subsp.* coloratum* increase mitochondrial oxygen consumption rate (OCR) in L6 cells and increase the expression of peroxisome proliferator-activated receptor c coactivator- (PGC-) 1a and silent mating type information regulation 2 homolog 1 (SIRT1), two major regulators of mitochondria function, in C2C12 cells, suggesting that this extract has great potential as a novel mitochondria-activating agent and could exert the antifatigue effect [38]. Jackson et al. try to understand how* Vitis vinifera* and its compound resveratrol could prevent muscle fatigue. Resveratrol has a protective effect against aging-induced oxidative stress in skeletal muscle, likely through the upregulation of manganese superoxide dismutase (MnSOD) activity, reducing hydrogen peroxide, and lipid peroxidation levels in muscle samples, but sarcopenia was not attenuated by resveratrol [51].

*Withania somnifera* (gradual escalating doses from 750 to 1250 mg/day) in humans has demonstrated muscle strengthening and lipid lowering [36].

In conclusion, there are several preclinical lines of evidence that botanical extracts, such as* Panax quinquefolius*,* A. marmelos *fruit,* Salvia sativa*,* Angelica sinensis*,* Phalaenopsis cornu-cervi*,* Cucurbita moschata*,* Withania somnifera*,* Acanthopanax senticosus*, deer antler extract, Tao-Hong-Si-Wu-Tang,* Rubus parvifolius* L., velvet antler extract,* Viscum album *subsp.* coloratum*, and* Vitis vinifera*, can reduce the muscle's fatigue, after intense exercise or simply in a condition of loss of muscle mass, as in sarcopenia (Table 4). Commonly, these properties are due to their antioxidant effects: in general, these plants reduce lipid peroxidation, lactic acid, and serum levels of ammonia and creatine kinase and increase liver and muscle glycogen. The only study found in human was that of Raut et al., in which supplementation with* Withania somnifera* (with gradual escalating doses from 750 to 1250 mg/day) seems to have good effects on antifatigue, but this is a preliminary study. Until today, the role of plants in antifatigue in clinical studies has been not deeply documented and so it is difficult to recommend particular supplementation.

### 3.5. Muscle Atrophy Prevention

This research has been carried out based on the keywords “skeletal muscle mass” and “atrophy” and “botanicals” or “plants” or “extracts”; 15 articles were sourced and 7 studies are taken into account. Among these, 4 are in in vitro settings and 2 are in animals and only one is in human (Table 6).

*Curcuma longa* can prevent muscle atrophy. It stimulates glucose-regulated protein 94 kDa (Grp94) expression in myogenic cells, whose levels decrease significantly in unloaded muscle, and it is involved in attenuation of myofiber atrophy in rats [43]. Also,* Camellia sinensis* extracts in rats appear to counteract the increased protein degradation (linked with its ability to downregulate key components of the ubiquitin proteasome proteolytic pathway) [25]. Instead,* Cichorium intybus* extract prevents skeletal muscle atrophy in vitro, probably increasing heat-shock protein-70 (Hsp-70) production and inhibiting the level of ceramide: Hsp-70, in fact, has a positive effect on reducing oxidative stress of cells and ceramide is involved in the regulation of cell death [45]. Also, chestnut sweet flour (rich in *γ*-tocopherol) protects from skeletal muscle cell atrophy, but this protection appears not to be due to a general antioxidant action, but maintaining cellular redox homeostasis through the regulation of NADPH oxidase, mitochondrial integrity [29]. Isoflavones are the most important phytochemicals in* Glycine max* for preventing muscle atrophy. These products could induce in vitro the expression of SIRT-1, a sirtuin that normally deacetylates p65, in order to reduce the activity of MuRF-1 related to muscle atrophy. Overall, they suppress MuRF-1 promoter activity and myotube atrophy induced by TNF-*α* in C2C12 myotubes [11]. However, a study performed by Choquette et al. demonstrated that, in postmenopausal women, only exercise, but not soy isoflavones (70 mg/day), could improve muscle strength and reduce risks of mobility impairments [57]. In addition, consumption of* Chlorella*, a unicellular green alga, could prevent age-related muscle atrophy in mice, because it contains various antioxidant substances, including carotenoids and vitamins and plastoquinone that has been shown to hold greater antioxidant properties.* Chlorella* contains also amino acids such as the brain chain amino acids (BCAA) valine, leucine, and isoleucine, which are important components of actin and myosin, the fundamental muscle proteins, and may be important in prevention of sarcopenia. Finally,* Chlorella* also prevents mitochondrial dysfunction [47].

In conclusion, it is clear that botanical extracts can prevent the atrophy of muscle, after intense exercise or simply in a condition of loss of muscle mass, as in sarcopenia. We considered several botanicals (*Curcuma longa*,* Camellia sinensis*,* Cichorium intybus*, chestnut sweet flour,* Glycine max*, and* Chlorella*): most of them have important antioxidant properties, which prevent muscle's atrophy. However, the only study made on human, using* Glycine max*, did not show positive results and so other researches are needed to substantiate the use of botanicals supplementation to prevent muscle atrophy.

### 3.6. Muscle Regeneration and Differentiation

This research has been carried out based on the keywords “skeletal muscle mass” and “regeneration” and “botanicals” or “plants” or “extracts”; 19 articles were sourced and 15 studies are taken into account. Among these, 4 are in in vitro settings, 7 in animals, and 3 in human and one is both in animals and in humans (Table 7). Nutraceutical compounds by* C. sinensis* in mice decrease myostatin and *β*-galactosidase and increase levels of markers of muscle; instead, in humans, they (7-day treatment with epicatechin at 1 mg/kg/day) increase hand grip strength and the ratio of plasma follistatin/myostatin [53] and regulate NF-*κ*B activity in regenerating muscle fibers [9].* Camellia* also induces changes in satellite cell number and it improves muscle recovery following a period of atrophy in old rats and decreases oxidative stress, but this is insufficient to improve muscle recovery following a period of atrophy [48]. Also, an increase in myogenin (due to a supplement of* Vitis vinifera* resveratrol extracts) served to stimulate differentiation to compensate for an impaired function of satellite cells (SCs) in the old muscles [26]. An article by Ballak et al., about resveratrol, says that this compound does not rescue the hypertrophic response and even reduces the number of satellite cells in hypertrophied muscle of mice [26]. Also,* Ferula hermonis* Boiss. and* Vitis vinifera* significantly increase muscle weight and enhance the growth of skeletal muscle fibers or fiber size (increase the fiber cross-sectional area of type IIA and IIB fibers) and nuclear number in order to enhance the growth of skeletal muscle [52, 58]. It is noteworthy that proanthocyanidins of* Vitis* have been used in a clinical trial. An increase of muscle mass and the improvement of several physical conditions have been observed in middle-aged women (with at least one menopausal symptom) treated with doses from 100 to 200 mg/d [60].* Broussonetia kazinoki* (*B*.* kazinoki*),* Corydalis turtschaninovii*, and Hachimijiogan, in vitro, promote myogenic differentiation through activation of key promyogenic kinase (p38 MAPK) or ERK1/2 and MyoD transcription activities (MyoD family transcription factors play a key role in promoting myoblast differentiation) without affecting the Akt signaling pathway [13–15]. Another in vitro study, performed by Poussard et al., indicated Oligopin, a* pine bark* extract, as natural antioxidant; in fact, with aging, oxidative stress produces disruption of cytoskeleton and phosphorylated heat-shock protein beta-1 (HSPB1) may help to repair injured structures. Furthermore, Oligopin prevents the stress-induced phosphorylation of HSPB-1 in human cells [27]. Curcumin (*Curcuma longa*) may modulate the entry into apoptosis during immobilization and stimulate initial steps of muscle regeneration, aging on proteins and enzyme such as proteasome chymotrypsin-like activity and proapoptotic smac/DIABLO protein levels, and apoptosome-linked caspase-9 activities [59]. Another study was performed in humans with* Withania somnifera*: it seems to improve muscle strength and endurance for the aged subjects and so it could be used in preventing sarcopenia (500–750 mg twice daily for three months) [5]. Finally, Kim et al. demonstrate that physical exercise combined with tea catechin supplementation (350 mL of a tea beverage fortified with 540 mg of catechins) had a beneficial effect on physical function measured by walking ability and muscle mass in women with sarcopenia [65].

Lastly, a very recent study [35] demonstrated in animal models that loquat (*Eriobotrya japonica*) leaf extract (LE) diminished the age-associated loss of grip strength and enhanced muscle mass and muscle creatine kinase (CK) activity. Histochemical analysis revealed that loquat (*Eriobotrya japonica*) leaf extract (LE) abrogated the age-associated decrease in cross-sectional area (CSA) and decreased the amount of connective tissue in the muscle of aged rats. Moreover, in order to investigate the mode of action, C2C12 murine myoblasts were used to evaluate the myogenic potential of LE. The expression levels of myogenic proteins (MyoD and myogenin) and functional myosin heavy chain (MyHC) were measured by western blot analysis. LE enhanced MyoD, myogenin, and MyHC expression. The changes in the expression of myogenic genes corresponded to an increase in the activity of CK, a myogenic differentiation marker. Finally, loquat (*Eriobotrya japonica*) leaf extract (LE) activated the Akt/mammalian target of rapamycin (mTOR) signaling pathway, which is involved in muscle protein synthesis during myogenesis. These findings suggest that loquat (*Eriobotrya japonica*) leaf extract (LE) attenuates sarcopenia by promoting myogenic differentiation and subsequently promoting muscle protein synthesis.

In conclusion, there are several preclinical lines of evidence for a variety of plants (*Camellia sinensis*,* Vitis vinifera*,* Ferula hermonis *Boiss.,* grape seed*,* Broussonetia kazinoki*,* Corydalis turtschaninovii*, Hachimijiogan,* pine bark*,* Curcuma longa*,* Withania somnifera*, and* Eriobotrya japonica*), but only four studies are available in humans: two of these were conducted with supplementation of* Camellia sinensis* products, one with* Withania somnifera* and one with* grape seed*. In particular, the use of* Withania somnifera* (50–750 mg twice a day) resulted in improving muscle strength in human and also the supplementation with 540 mg of catechin from* Camellia sinensis* induced positive physical improvement. The second study demonstrated an improvement in grip strength, but it was only an experimental study with 25 mg of pure EGCG. Finally, the clinical trial with* grape seed* (100–200 mg/d) seemed to increase muscle mass and improve other physical conditions during menopause. For muscle regeneration, the main studies to take into account were those performed by Kim et al. and by Mishra et al., in which sarcopenic subjects have been enrolled. However, it is clear that the supplementation with EGCG should be complementary to appropriate physical exercise in order to reach the beneficial effects on muscle mass and that further studies are needed also for* Withania* supplementation.

## 4. Discussion

Currently, only diet and exercise are recognized as an effective means to counteract loss of muscle [53]. Regarding exercise, it is important to note that exercise-induced muscle damage (EIMD) can be caused by eccentric type or unaccustomed (novel) exercise and results in decrements in muscle force production, development of delayed-onset muscle soreness (DOMS) and swelling, rise in passive tension, and an increase in blood intramuscular proteins [66].

Delayed-onset muscle soreness is generally considered a hallmark sign of EIMD [67], and it is thought that DOMS is partially related to direct muscle fiber damage, and its magnitude appears to vary with the type, duration, and intensity of exercise [68].

The inflammatory response to EIMD results in the release into blood of reactive species from both neutrophils and macrophages and an array of cytokines from the injured muscle including tumor necrosis factor- (TNF-) *α*, interleukin- (IL-) 1*β*, and IL-6, which contribute to low-grade systemic inflammation and oxidative stress [69]. The proinflammatory and prooxidant response can provoke secondary tissue damage [70], thus prolonging the regenerative process, which is generally characterized by restoration of muscle strength and resolution of inflammation [70]. All these phenomena must be avoided in elderly sarcopenic subjects and so it is critical in this population to better preserve skeletal muscle and muscle function.

In this review, we focused our attention on effects of several botanicals on growth and health of muscle and we divided these effects into five categories: anti-inflammation, muscle damage prevention, antifatigue, muscle atrophy prevention, and muscle regeneration and differentiation.

To date, although the animal studies and in vitro studies are numerous and promising, studies in humans evaluating the effectiveness of anti-inflammatory and antioxidant activities of botanicals on welfare of skeletal muscle are still very few.

Although only relatively few human studies have been published on the potential use of botanicals for the prevention and treatment of muscle function, the present review is important because it highlights the need of continued efforts to find effective treatment of this debilitating condition. The available results, in particular considering human studies, suggest that the botanicals that may be potentially useful dietary supplements to prevent loss of muscle mass and function are curcumin from* Curcuma longa*, alkaloids and steroidal lactones from* Withania somnifera* (Solanaceae), catechins from* Camellia sinensis*, proanthocyanidin of* grape seeds*, and gingerols and shogaols from* Zingiber officinale*.

It should be noted that this review is not claiming that the use of these botanicals has been proven to prevent and treat loss of muscle mass and muscle function, but we believe that early and preliminary observations are promising. Further researches will support the use of these botanicals in the management of age-related muscle dysfunction and this may open the possibility of treating age-related loss of muscle mass and function with supplements.

## Figures and Tables

**Table 1:** Summary of methodology.

Step	General activities	Specific activities
Step 1	Configuration of a working group	Selection of three operators skilled in clinical nutrition: (i) One as methodological operator (ii) Two as clinical operators

Step 2	Formulation of the revision question	Evaluation of the state of the art in metabolic and nutritional disorders of sarcopenia and their treatment with botanicals

Step 3	Identification of relevant studies on PubMed	(a) Identification of the key words (sarcopenia, nutrients, and dietary supplement), allowing the definition of the interest field of the documents to be searched, grouped in inverted commas (“…”), and used separately or in combination (b) Use of the Boolean (a data type with only two possible values: true and false) AND operator, which allows the establishment of logical relations among concepts (c) Research modalities: advanced search (d) Limits: papers published in the last 20 years; in vitro, animal, and humans studies; languages: English (e) Manual search performed by the senior researchers experienced in clinical nutrition through the revision of reviews and individual articles on sarcopenia in the elderly, published in journals qualified in the Index Medicus

Step 4	Analysis and presentation of the outcomes	The data extrapolated from the revised studies was investigated in the form of a narrative review of the reports and was collocated in tables

**Table 2:** Effects on skeletal muscle for each botanical.

Effect	Botanicals	Physiology	Study	Authors
Downregulation of LPS-induced COX-2 and iNOS expression	Korean *Citrus aurantium* L.	10, 50, 75, and 100 *µ*g/mL	Rat skeletal muscle cells	Kim et al., 2012 [7]

Suppression or inhibition of NF-*κ*B	Korean *Citrus aurantium* L.	10, 50, 75, and 100 *µ*g/mL	Rat skeletal muscle cells	Kim et al., 2012 [7]
	*Eugenia punicifolia*	2 mg/mL	Male mdx dystrophic mice	Leite et al., 2010 [8]
	*Camellia sinensis*	0.25% or 0.5% green tea extract, at the age of 42 days	C57BL/6J and mdx mice	Evans et al., 2010 [9]

Increase of NF-*κ*B	*Panax ginseng*	DS 20, 60, and 120 mg/kg	Rats	Yu et al., 2014 [10]

Induction of the phosporylation of AMPK	*Glycine max*	100 *µ*M	C2C12 myotubes	Hirasaka et al., 2013 [11]

Decrease of MURF-1 promoter activity	*Glycine max*	100 *µ*M	C2C12 myotubes	Hirasaka et al., 2013 [11]

Suppression of LPS-induced phosphorylation of the MAPKs (JNK, ERK, and p38 MAPK pERK)	Korean *Citrus aurantium* L.	10, 50, 75, and 100 *µ*g/mL	Rat skeletal muscle cells	Kim et al., 2012 [7]
	*Eugenia punicifolia*	100 *µ*g/mL	Mouse myoblastoma cells (C2C12)	Leite et al., 2014 [12]

Increase of ERK1/2 activity	Hachimijiogan (HJG)	HJG treatment (1–200 *μ*g/mL)	Murine skeletal cells	Takeda et al., 2015 [13]

Activation of p38 MAPK signaling	*Broussonetia kazinoki* (pp38)	KP in 2% HS for 48 h, 10–1000 nM	C2C12 and 10T1/2 cells	Hwang et al., 2015 [14]
	*Corydalis turtschaninovii* (p-p38)	Various concentrations of THP	C2C12 myoblasts and fibroblast 10T1/2	Lee et al., 2014 [15]

Increase of myogenin	*Eugenia punicifolia* (17.5-kDa)	2 mg/mL	Male mdx dystrophic mice	Leite et al., 2010 [8]

Increased expression of MHC, myogenin, and Troponin-T	*Broussonetia kazinoki*	KP in 2% HS for 48 h, 10–1000 nM	C2C12 and 10T1/2 cells	Hwang et al., 2015 [14]
	*Corydalis turtschaninovii*	Various concentrations of THP	C2C12 myoblasts and fibroblast 10T1/2	Lee et al., 2014 [15]

Decrease of the expression of TNF-*α*	Korean *Citrus aurantium* L.	10, 50, 75, and 100 *µ*g/mL	Rat skeletal muscle cells	Kim et al., 2012 [7]
	*Vitis vinifera*	100 *μ*M	Mouse C2C12 cells	Wang et al., 2014 [16]
	*Panax notoginseng*	5 mg of Rg1	Healthy young men (*N* = 26)	Hou et al., 2015 [17]
	*Phlebodium decumanum*	5 capsules of 400 mg (250 mg of leaf extract and 150 mg of rhizome extract)	Amateur athletes (*N* = 40)	Díaz-Castro et al., 2012 [18]
	*Eugenia punicifolia*	2 mg/mL	Male mdx dystrophic mice	Leite et al., 2010 [8]
	Coffee	The same amount of drink in control and coffee group for 4 weeks	C57BL/6 mice	Guo et al., 2014 [19]
	*Camellia sinensis*	0.5% w/w in diet for 3 weeks after downhill running	Mice	Haramizu et al., 2013 [20]
	*Rubus parvifolius* L. (RPL)	40 mg/kg and 20 mg/kg	Five-week-old male	Chen et al., 2013 [21]
	*Glycine max*	100 *µ*M	C2C12 myotubes	Hirasaka et al., 2013 [11]

Increase in sTNF-RII	*Phlebodium decumanum*	5 capsules of 400 mg (250 mg of leaf extract and 150 mg of rhizome extract)	Amateur athletes (*N* = 40)	Díaz-Castro et al., 2012 [18]

Decrease of IL-1*α*	Coffee	The same amount of drink in control and coffee group for 4 weeks	C57BL/6 mice	Guo et al., 2014 [19]

Decrease of IL-6	Korean *Citrus aurantium* L.	10, 50, 75, and 100 *µ*g/mL	Rat skeletal muscle cells	Kim et al., 2012 [7]
	Curcumin (at 24 h)	2.5 g twice daily	Men (*N* = 17)	Nicol et al., 2015 [22]
	Coffee	The same amount of drink in control and coffee group for 4 weeks	C57BL/6 mice	Guo et al., 2014 [19]
	*Rubus parvifolius* L. (RPL)	40 mg/kg and 20 mg/kg	Five-week-old male	Chen et al., 2013 [21]

Increase of interleukin-6 (IL-6)	Curcumin (at 0 h and 48 h)	2.5 g twice daily	Men (*N* = 17)	Nicol et al., 2015 [22]

Decrease of IL-8	Curcumin	1 g twice daily (corresponding to 200 mg curcumin twice a day)	Healthy, moderately active male (*N* = 20)	Drobnic et al., 2014 [23]

Decrease of IL-1*β*	*Eugenia punicifolia* (17.5-kDa)	2 mg/mL	Male mdx dystrophic mice	Leite et al., 2010 [8]
	*Panax ginseng*	DS	Rats	Yu et al., 2014 [10]
	*Camellia sinensis*	0.5% w/w in diet for 3 weeks after downhill running	Mice	Haramizu et al., 2013 [20]

Increase of IL-10	*Panax notoginseng*	5 mg of Rg1	Healthy young men (*N* = 26)	Hou et al., 2015 [17]
	*Panax ginseng*	DS 20, 60, and 120 mg/kg	Rats	Yu et al., 2014 [10]

Decrease of MCP-1	*Camellia sinensis*	0.5% w/w in diet for 3 weeks after downhill running	Mice	Haramizu et al., 2013 [20]

Decreased MnSOD (only at high dose)	*Panax ginseng*	DS 20, 60, and 120 mg/kg	Rats	Yu et al., 2014 [10]

Decrease of the expression of cleaved caspase-3	Korean *Citrus aurantium* L.	100 *µ*g	Rat skeletal muscle cells	Kim et al., 2013 [24]
	*Eugenia punicifolia*	2 mg/mL	Male mdx dystrophic mice	Leite et al., 2010 [8]

Increased expression of antioxidant enzymes, such as GPx (not at high dose) and GCS	*Panax ginseng*	DS 20, 60, and 120 mg/kg	Rats	Yu et al., 2014 [10]
	*Phlebodium decumanum* (only GPx)	5 capsules of 400 mg (250 mg of leaf extract and 150 mg of rhizome extract)	Amateur athletes (*N* = 40)	Díaz-Castro et al., 2012 [18]

Increase of MMP-9 and MMP-2	*Eugenia punicifolia*	100 *µ*g/mL	Mouse myoblastoma cells (C2C12)	Leite et al., 2014 [12]

Reduced MMP-9	*Eugenia punicifolia*	2 mg/mL	C57BL/10 mice	Leite et al., 2014 [12]

Reduced MMP-9 and MMP-2	*Eugenia punicifolia*	2 mg/mL	Male mdx dystrophic mice	Leite et al., 2010 [8]

Increase of citrate synthase (CS) activity	*Panax notoginseng*	5 mg of Rg1	Healthy young men (*N* = 26)	Hou et al., 2015 [17]

Attenuation of the increases in mRNAs encoding Ly6G and CD68 observed at 24 h after downhill running	*Camellia sinensis*	0.5% w/w in diet for 3 weeks after downhill running	Mice	Haramizu et al., 2013 [20]

Increase of p27 and pAkt	*Eugenia punicifolia*	100 *µ*g/mL	Mouse myoblastoma cells (C2C12)	Leite et al., 2014 [12]
	*Corydalis turtschaninovii* (only pAkt)	Various concentrations of THP	C2C12 myoblasts and fibroblast 10T1/2	Lee et al., 2014 [15]

Reduction of Cyclin D1	*Eugenia punicifolia*	100 *µ*g/mL	Mouse myoblastoma cells (C2C12)	Leite et al., 2014 [12]

Increased expression of pAkt and pFoxO3a	*Camellia sinensis*	10–150 *μ*M	C2C12 myotubes	Mirza et al., 2014 [25]

Decrease in MPO activity	*Camellia sinensis*	0.5% w/w in diet for 3 weeks after downhill running	Mice	Haramizu et al., 2013 [20]

Decreased caspase-3 expression	*Vitis vinifera*	0.4 mg per gram body mass per day	Mice (male C57BL/6J mice)	Ballak et al., 2015 [26]

Upregulation of phosphorylation of Akt, p70S6K, mTOR, and 4E-BP1	*Vitis vinifera*	100 *μ*M	Mouse C2C12 cells	Wang et al., 2014 [16]
	Coffee	Coffee solution 10, 30, 50, and 100 *µ*g/mL	Mouse myosatellite cells	Guo et al., 2014 [19]

Prevention of HSPB1 phosphorylation	*Pinus pinaster*	0.05 mg/mL	Human muscle satellite cells	Poussard et al., 2013 [27]

Decrease in FoxO1 protein and promotion of FoxO1 phosphorylation	*Vitis vinifera*	100 *μ*M	Mouse C2C12 cells	Wang et al., 2014 [16]

Decreased MURF-1 and MAFbx	*Camellia sinensis*	10–150 *μ*M	C2C12 myotubes	Mirza et al., 2014 [25]

Increased MURF-1	Go-sha-jinki-Gan (GJG) (only PGC-1*α*)	4% (w/w)	Male SAMP8, SAMR1 mice	Kishida et al., 2015 [28]

Increase of the expression of MAFbx/atrogin1	Chestnuts flour	Polyphenols (100 nM) or tocopherols (100 nM)	C2C12 myotube cells	Frati et al., 2014 [29]

Decreased expression of proteasomes 20S and 19S	*Camellia sinensis*	10–150 *μ*M	C2C12 myotubes	Mirza et al., 2014 [25]

Decreased peak CK serum or activity	Curcumin	150 mg before and 12 h after each eccentric exercise	Untrained young men	Tanabe et al., 2015 [30]
	Curcumin	2.5 g twice daily	Men (*N* = 17)	Nicol et al., 2015 [22]
	Curcumin	200 mg/kg/day	Male Wistar rats	Boz et al., 2014 [31]

Decrease plasma-serum ammonia levels	Pumpkin (*Cucurbita moschata*) fruit	0, 50, 100, and 250 mg/kg/day for 14 days	Male ICR mice	Wang et al., 2012 [32]
	*Angelica sinensis*	0.41 g/kg/day (Ex-AS1) and 2.05 g/kg/day (Ex-AS5), 6 weeks	Male ICR strain mice	Yeh et al., 2014 [33]
	*Rubus parvifolius* L. (RPL)	40 mg/kg and 20 mg/kg	Five-week-old male	Chen et al., 2013 [21]

Increase in blood creatine kinase	*Zingiber officinale* Roscoe	4 g of ginger once a day for 5 days	20 non-weight trained participants	Matsumura et al., 2015 [34]
	*Eriobotrya japonica*	50 mg/kg/day	Young (5-month-old) and aged (18-19-month-old) rats	Sung et al., 2015 [35]

Increase in serum creatinine	Ashwagandha (*Withania somnifera*) (WS)	750 mg/day × 10 days; 1000 mg/day × 10 days; 1250 mg/day × 10 days	Eighteen apparently healthy volunteers	Raut et al., 2012 [36]

Decrease of serum creatine kinase activity	Pumpkin (*Cucurbita moschata*) fruit	0, 50, 100, and 250 mg/kg/day for 14 days	Male ICR mice	Wang et al., 2012 [32]
	*Salvia officinalis *	100, 200, and 300 mg/kg BW	50 rats	JiPing, 2011 [37]
	*Angelica sinensis*	0.41 g/kg/day (Ex-AS1) and 2.05 g/kg/day (Ex-AS5), 6 weeks	Male ICR strain mice	Yeh et al., 2014 [33]
	*Camellia sinensis*	0.25% or 0.5% green tea extract at the age of 42 days	C57BL/6J and mdx mice	Evans et al., 2010 [9]
	*Withania somnifera*	500 mg of the whole root extract twice daily; 750 mg twice daily	35 individuals	Mishra and Trikamji, 2013 [5]

Decrease in plasma lactate or lactic acid	Korean mistletoe (*Viscum album* subsp. *coloratum*)	KME at 400 or 1000 mg/(kg·d) for 1 week and 25, 40, 200, and 400 mg/kg	ICR mice	Jung et al., 2012 [38]
	*Aegle marmelos* (L.) Corr.	100, 200, and 400 mg/kg BW for 21 d	BALB/c mice	Nallamuthu et al., 2014 [39]
	Pumpkin (*Cucurbita moschata*) fruit	0, 50, 100, and 250 mg/kg/day for 14 days	Male ICR mice	Wang et al., 2012 [32]
	Tao-Hong-Si-Wu-Tang (THSWT)	5, 10, and 20 mL/ kg body weight for 28 days	32 male mice	Li et al., 2013 [40]
	*Rubus parvifolius* L. (RPL)	40 mg/kg and 20 mg/kg	Five-week-old male	Chen et al., 2013 [21]
	*Angelica sinensis*	0.41 g/kg/day (Ex-AS1) and 2.05 g/kg/day (Ex-AS5), 6 weeks	Male ICR strain mice	Yeh et al., 2014 [33]

Increase in LDH and lactic acid	*Panax ginseng*	10 mg/kg	Rat	Tan et al., 2013 [41]
	*Acanthopanax senticosus* (LDH)	500 mg/kg and 200 mg/kg; 280 mg/kg or 70 mg/kg; 70 mg/kg or 280 mg/kg	Five-week-old male ICR mice	Huang et al., 2011 [42]
	*Rubus parvifolius* L. (RPL)	40 mg/kg and 20 mg/kg	Five-week-old male	Chen et al., 2013 [21]

Decreased myoglobin levels	Curcumin	200 mg/kg/day	Male Wistar rats	Boz et al., 2014 [31]

Decreased MDA levels in liver tissue	Curcumin	200 mg/kg/day	Male Wistar rats	Boz et al., 2014 [31]
	*Aegle marmelos* (L.) Corr.	100, 200, and 400 mg/kg BW for 21 days	BALB/c mice	Nallamuthu et al., 2014 [39]
	*Curcuma longa*	20–40 *μ*g kg^−1^ of curcumin	Wistar rats (*n* = 130)	Vitadello et al., 2014 [43]

Increased MDA	*Panax ginseng*	10 mg/kg	Rat	Tan et al., 2013 [41]

Increased availability of serum free fatty acid	*Aegle marmelos* (L.) Corr.	400 mg/kg BW for 21 days	BALB/c mice	Nallamuthu et al., 2014 [39]

Decreased level of TG	*Acanthopanax senticosus*	500 mg/kg and 200 mg/kg; 280 mg/kg or 70 mg/kg; 70 mg/kg or 280 mg/kg	Five-week-old male ICR mice	Huang et al., 2011 [42]
	*Rubus parvifolius* L. (RPL)	40 mg/kg and 20 mg/kg	Five-week-old male	Chen et al., 2013 [21]
	Ashwagandha (*Withania somnifera*) (WS)	750 mg/day × 10 days; 1000 mg/day × 10 days; 1250 mg/day × 10 days	Eighteen apparently healthy volunteers	Raut et al., 2012 [36]

Decrease in glucose and insulin	*Panax notoginseng*	5 mg of Rg1	Healthy young men (*N* = 26)	Hou et al., 2015 [17]

Increase in blood glucose	Pumpkin (*Cucurbita moschata*) fruit	0, 50, 100, and 250 mg/kg/day for 14 days	Male ICR mice	Wang et al., 2012 [32]
	*Angelica sinensis*	0.41 g/kg/day (Ex-AS1) and 2.05 g/kg/day (Ex-AS5), 6 weeks	Male ICR strain mice	Yeh et al., 2014 [33]

Increase in citrate synthase (CS) activity	*Panax notoginseng*	5 mg of Rg1	Healthy young men (*N* = 26)	Hou et al., 2015 [17]

Rate of glycogen accumulation	*Panax notoginseng*	5 mg of Rg1	Healthy young men (*N* = 26)	Hou et al., 2015 [17]

Increase in glycogen content of liver and muscle	*Aegle marmelos* (L.) Corr.	100, 200, and 400 mg/kg BW for 21 d	BALB/c mice	Nallamuthu et al., 2014 [39]
	Pumpkin (*Cucurbita moschata*) fruit	0, 50, 100, and 250 mg/kg/day for 14 days	Male ICR mice	Wang et al., 2012 [32]
	*Panax ginseng*	10 mg/kg	Rat	Tan et al., 2013 [41]
	Tao-Hong-Si-Wu-Tang (THSWT)	5, 10, and 20 mL/kg body weight for 28 days	32 male mice	Li et al., 2013 [40]
	*Angelica sinensis*	0.41 g/kg/day (Ex-AS1) and 2.05 g/kg/day (Ex-AS5), 6 weeks	Male ICR strain mice	Yeh et al., 2014 [33]
	*Rubus parvifolius* L. (RPL)	40 mg/kg and 20 mg/kg	Five-week-old male	Chen et al., 2013 [21]

Increase in cholinesterase (ChE)	*Salvia officinalis *	100, 200, and 300 mg/kg BW	50 rats	JiPing, 2011 [37]

Upregulation of HSP70 mRNA levels or induction of the expression of Hsp-70	*Rhodiola rosea*	10 *μ*g/mL of Rhodiolife	Murine skeletal muscle cells	Hernández-Santana et al., 2014 [44]
	*Cichorium intybus* (Cii)	5, 10, 25, and 50 *µ*g/mL	C2C12 myoblast	Lee et al., 2013 [45]

Downregulation of Hsp-70	*Aegle marmelos* (L.) Corr.	100, 200, and 400 mg/kg BW for 21 days	BALB/c mice	Nallamuthu et al., 2014 [39]

Prevention of calpain upregulation	*Pinus pinaster*	Oligopin (0.05 mg/mL)	Cultured human skeletal muscle satellite cells	Dargelos et al., 2010 [46]

Inhibition of the level of ceramide	*Cichorium intybus* (Cii)	5, 10, 25, and 50 *µ*g/mL	C2C12 myoblast	Lee et al., 2013 [45]

Suppression or mitigation of the increases in plasma CPK, AST, ALT, and MDA levels after downhill running	*Camellia sinensis*	0.5% w/w in diet for 3 weeks after downhill running	Mice	Haramizu et al., 2013 [20]
	*Chlorella* (only CPK)	1% *Chlorella*-supplemented diet (CSD group)	Transgenic mice	Nakashima et al., 2014 [47]

Reduction of the levels of carbonylated protein	*Camellia sinensis*	0.5% w/w in diet for 3 weeks after downhill running	Mice	Haramizu et al., 2013 [20]
	*Curcuma longa*	20–40 *μ*g kg^−1^ of curcumin	Wistar rats (*n* = 130)	Vitadello et al., 2014 [43]
	*Camellia sinensis*	GTE (50 mg/kg body weight)	Sixty male rats	Alway et al., 2015 [48]

Attenuation of hydrogen peroxide concentration	*Curcuma longa*	3 mg	Male C57BL/6 mice	Kawanishi et al., 2013 [49]

Attenuation of NADPH-oxidase mRNA expression	*Curcuma longa*	3 mg	Male C57BL/6 mice	Kawanishi et al., 2013 [49]

Attenuation of F4/80 mRNA expression	*Curcuma longa*	3 mg	Male C57BL/6 mice	Kawanishi et al., 2013 [49]

Counteraction of the increase of BiP, ATF4, XBP1u, and XBP1s mRNA	*Camellia sinensis*	Green tea extract (0.5% w/vol)	Twelve-week-old female C57BL/6J mice	Rodriguez et al., 2014 [50]

Increase in the mitochondrial oxygen consumption rate	Korean mistletoe (*Viscum album* subsp. *coloratum*)	6 *µ*g/mL	L6 cells and C2C12 cells, mice	Jung et al., 2012 [38]

Increase of the expression of peroxisome proliferator-activated receptor coactivator- (PGC-) 1*α* and SIRT-1	Korean mistletoe (*Viscum album* subsp. *coloratum*)	6 *µ*g/mL	L6 cells and C2C12 cells, mice	Jung et al., 2012 [38]
	*Vitis vinifera*	0.05% trans-resveratrol for 10 months	Middle-aged (18 months) C57/BL6 mice	Jackson et al., 2011 [51]
	*Glycine max*	100 *µ*M	C2C12 myotubes	Hirasaka et al., 2013 [11]
	*Vitis vinifera*	125 mg/kg/day	Thirty-six male rats	Bennett et al., 2013 [52]

Decrease of PGC-1*α* expression	Go-sha-jinki-Gan (GJG) (only PGC-1*α*)	4% (w/w)	Male SAMP8, SAMR1 mice	Kishida et al., 2015 [28]

Decrease of BUN	*Aegle marmelos* (L.) Corr.	400 mg/kg BW for 21 days	BALB/c mice	Nallamuthu et al., 2014 [39]
	Pumpkin (*Cucurbita moschata*) fruit	0, 50, 100, and 250 mg/kg/day for 14 days	Male ICR mice	Wang et al., 2012 [32]
	Tao-Hong-Si-Wu-Tang (THSWT)	5, 10, and 20 mL/kg body weight for 28 days	32 male mice	Li et al., 2013 [40]
	*Acanthopanax senticosus*	500 mg/kg and 200 mg/kg; 280 mg/kg or 70 mg/kg; 70 mg/kg or 280 mg/kg	Five-week-old male ICR mice	Huang et al., 2011 [42]
	Ashwagandha (*Withania somnifera*) (WS)	750 mg/day × 10 days; 1000 mg/day × 10 days; 1250 mg/day × 10 days	Eighteen apparently healthy volunteers	Raut et al., 2012 [36]

Increase of SOD and catalase	*Aegle marmelos* (L.) Corr.	100, 200, and 400 mg/kg BW for 21 d	BALB/c mice	Nallamuthu et al., 2014 [39]
	*Panax ginseng* (only SOD)	10 mg/kg	Rat	Tan et al., 2013 [41]
	*Salvia officinalis* (SOD and GSHPx)	100, 200, and 300 mg/kg BW	50 rats	JiPing, 2011 [37]

Upregulation of GLUT-4 and AMPK-1*α*	*Aegle marmelos* (L.) Corr.	100, 200, and 400 mg/kg BW for 21 d	BALB/c mice	Nallamuthu et al., 2014 [39]

Decrease in SUN levels	*Rubus parvifolius* L. (RPL)	40 mg/kg and 20 mg/kg	Five-week-old male	Chen et al., 2013 [21]

Increase of Grp94 protein	*Curcuma longa*	20–40 *μ*g kg^−1^ of curcumin	Wistar rats (*n* = 130)	Vitadello et al., 2014 [43]

Decrease in myostatin and *β*-galactosidase	*Camellia sinensis*	1 mg/kg b.i.d.	Young and old C57BL/6 male mice	Gutierrez-Salmean et al., 2014 [53]

Increase in the ratio of plasma follistatin/myostatin	*Camellia sinensis*	25 mg of pure Epi (~1 mg/kg/day)	Human subjects (*n* = 6)	Gutierrez-Salmean et al., 2014 [53]

Decrease in cross-sectional area (CSA)	*Eriobotrya japonica*	50 mg/kg/day	Young (5-month-old) and aged (18-19-month-old) rats	Sung et al., 2015 [35]

**Table 3:** Botanicals with anti-inflammatory effects on skeletal muscle.

Paper	Botanical	Compound	Model	Physiology	Main results
In vitro					

Kim et al., 2012 [7]	Korean *Citrus aurantium* L.	Flavonoids (hesperidin, nobiletin, and naringin)	Rat skeletal muscle cells	Flavonoids 10, 50, 75, and 100 *µ*g/mL	Decrease in the production of inducible nitric oxide synthase, cyclooxygenase-2, TNF-*α*, and IL-6.

Kim et al., 2013 [24]	Korean *Citrus aurantium* L.	Flavonoids (naringin, hesperidin, poncirin, isosinnesetin, and hexamethoxyflavone)	Rat skeletal muscle cells	100 *µ*g	Protection of cell-structure related proteins and decrease in level of cleaved caspase-3.

Leite et al., 2014 [12]	*Eugenia punicifolia*	Pentacyclic triterpenes (barbinervic acid)	Mouse myoblastoma cells (C2C12)	Ep-CM 100 *µ*g/mL	Reduction of C2C12 cell density and proliferation. Increase of metalloproteases activity: MMP-9 (128 ± 14%, *p* < 0.005) and MMP-2 (110 ± 18%, *p* < 0.005).

Wang et al., 2014 [16]	*Vitis vinifera*	Resveratrol (3,5,40-trihydroxystilbene)	Mouse C2C12 cells	Resveratrol 100 *μ*M	Counteraction of TNF-*α* induced muscle protein loss and reversion of declining expression of Akt, mTOR, p70S6K, 4E-BP1, and FoX01.

Guo et al., 2014 [19]	Coffee	Chlorogenic acid, anhydrous caffeine, and polyphenols	Mouse myosatellite cells	Coffee solution 10, 30, 50, and 100 *µ*g/mL	Increase in cell proliferation rate, enhancement of the DNA synthesis of the proliferating satellite cells, and increase of the activation level of Akt.

Animals					

Yu et al., 2014 [10]	*Panax ginseng*	Dammarane steroids (DS)	Rats	DS 20, 60, and 120 mg/kg	Anti-inflammatory effects on skeletal muscle following muscle-damaging exercise.

Kishida et al., 2015 [28]	Go-sha-jinki-Gan (GJG)	Paeoniflorin, loganin, and total alkaloids	Male SAMP8, SAMR1 mice	GJG 4% (w/w)	Reduction of the loss of skeletal muscle mass and amelioration of the increase in slow skeletal muscle fibers.

Guo et al., 2014 [19]	Coffee	Coffee bean, chlorogenic acid, anhydrous caffeine, and polyphenols	C57BL/6 mice	The same amount of drink in control and coffee group for 4 weeks	Improvement in grip strength; faster regeneration of injured skeletal muscles. Decrease in the levels of interleukins.

Leite et al., 2010 [8]	*Eugenia punicifolia*	Dichloromethane fraction	Male mdx dystrophic mice	Ep-CM 2 mg/mL	Reduction of MMP-9 (62 ± 12%, *p* < 0.005) and MMP-2 (58 ± 10%, *p* < 0.005) activities. Reduction of TNF-*α* production (42 ± 9%, *p* < 0.01) and NF-*κ*B expression (48 ± 7%, *p* < 0.005).

Leite et al., 2014 [12]	*Eugenia punicifolia*	Pentacyclic triterpenes (barbinervic acid)	C57BL/10 mice	Ep-CM 2 mg/mL	Reduction of MMP-9 activity (35 ± 7%, *p* < 0.05) but difference concerning MMP-2 activity in the muscular lesion; reduction of the inflammatory lesion area.

Boz et al., 2014 [31]	Curcumin	Curcumin	Male Wistar rats	200 mg/kg/day	Decrease of CK activity (*p* > 0.05) and significant decrease of myoglobin levels (*p* < 0.05).

Humans					

Díaz-Castro et al., 2012 [18]	*Phlebodium decumanum*	Polyphenols, terpenoids, and xavonoids	Amateur athletes (*N* = 40)	5 capsules of 400 mg (250 mg of leaf extract and 150 mg of rhizome extract)	Reduction of oxidative stress (*p* < 0.0001). Reduction in the inflammatory response. Decrease of TNF-*α* before and after the high-intensity exercise. Increase in sTNF-RII.

Hou et al., 2015 [17]	*Panax notoginseng*	Ginsenosides Rg1	Healthy young men (*N* = 26)	5 mg of Rg1	Increase in exercise time to exhaustion (Rg1 38.3 ± 6.7 min versus placebo 31.8 ± 5.0 min). Improvement in meal tolerance during recovery (*p* < 0.05).

Black et al., 2010 [54]	*Zingiber officinale* Roscoe	Gingerols and shogaols	Individuals (*N* = 28)	2 g of ginger after exercise	Postexercise reduction in arm pain the following day (13%; −5.9 ± 8 mm).

Black et al., 2010 [54]	*Zingiber officinale* Roscoe	Gingerols and shogaols	34 participants in study 1 40 participants in study 2	2 g for 11 consecutive days after exercise	Decrease in pain-intensity ratings 24 hours after eccentric exercise in both studies (*p* < 0.05).

Pumpa et al., 2013 [55]	*Panax notoginseng*	Saponins (ginsenosides)	Well-trained male volunteers (*N* = 20)	4 g of *P. notoginseng*	Decrease in IL-6 24 h after the downhill run (placebo). Decrease in TNF-*α* 24 h after the downhill run (placebo).

Drobnic et al., 2014 [23]	Curcumin	Phytosome delivery system (Meriva)	Healthy, moderately active male (*N* = 20)	1 g twice daily (corresponding to 200 mg curcumin twice a day)	Significant decrease in pain intensity for the right and left anterior thigh (4.4 ± 2.5 and 4.4 ± 2.4, *p* < 0.05). Lower increase in hsPCR levels at 24 hours (116.2%). Lower increase of IL-8 levels at 2 hours (196.8 ± 66.1 pg/mL, *p* < 0.05).

Nicol et al., 2015 [22]	Curcumin	Curcuminoids	Men (*N* = 17)	2.5 g twice daily	Moderate-to-large reduction in pain during single-leg squat (VAS scale −1.4 to −1.7; 90% CL: ±1.0), gluteal stretch (−1.0 to −1.9; ±0.9), and squat jump (−1.5 to −1.1; ±1.2) and reduction in creatine kinase activity (−22–29%; ±21-22%). Increase in IL-6 concentrations at 0 h (31%; ±29%) and 48 h (32%; ±29%), but decrease in IL-6 at 24 h relative to postexercise period (−20%; ±18%).

Tanabe et al., 2015 [30]	Curcumin	Curcuminoids (Theracurmin)	Untrained young men (*N* = 14)	150 mg before and 12 h after each eccentric exercise	Faster recovery of maximum voluntary contraction torque (e.g., 4 days after exercise: −31 ± 13% versus −15 ± 15%), lower peak serum CK activity (peak: 7684 ± 8959 IU/L versus 3398 ± 3562 IU/L, *p* < 0.05). No significant changes in IL-6 and TNF-*α* after exercise.

**Table 4:** Botanicals with counterbalancing muscle damage effects.

Paper	Botanical	Compound	Model	Physiology	Main results
In vitro					

Hernández-Santana et al., 2014 [44]	*Rhodiola rosea*	RR extracts: rosavins and salidroside	Murine skeletal muscle cells	1–100 *µ*g/mL and others	Upregulation of HSP70 mRNA levels and enhancement of the expression by exposure to H_2_O_2_ (*p* < 0.05). Maintenance of HSP70 protein levels in pretreated cell cultures compared to controls (−50%).

Dargelos et al., 2010 [46]	*Pinus pinaster*	Polyphenols	Cultured human skeletal muscle satellite cells	Oligopin (0.05 mg/mL)	Restoration of cell viability (55.2 ± 3.2% versus 42.3 ± 4.8% in H_2_O_2 _ treated cells). Abolishment of H_2_O_2 _ induced apoptotic cell death.

Animals					

Haramizu et al., 2013 [20]	*Camellia sinensis*	Catechins: epigallocatechin gallate, epigallocatechin, epicatechin gallate, epicatechin, gallocatechin, and gallocatechin gallate	Mice	0.5% w/w in diet for 3 weeks after downhill running	Mitigation of the running-induced decrease in voluntary wheel-running activity by 35%. Maintenance of endurance running capacity (214 ± 9 versus 189 ± 10 min, *p* < 0.05).

Kawanishi et al., 2013 [49]	*Curcuma longa*	Curcumin	Male C57BL/6 mice	3 mg	Decrease of hydrogen peroxide concentration and NADPH-oxidase mRNA expression (*p* < 0.05).

Rodriguez et al., 2014 [50]	*Camellia sinensis *	Green tea extracts	Twelve-week-old female C57BL/6J mice	Green tea extract (0.5% w/vol)	Decrease of BiP, ATF4, XBP1u, and XBP1s mRNA. No activity on CHOP mRNA.

Humans					

Shanely et al., 2014 [56]	*Rhodiola rosea *	Rosavin, salidroside, syringin, triandrin, and tyrosol	55 subjects (48 completing all aspects of the study)	600 mg/day for 30 days prior to, on the day of, and after 7 days of the marathon	No effects on DOMS increased (*p* = 0.700).

Pumpa et al., 2013 [55]	*Panax notoginseng*	Saponins (ginsenosides)	Twenty well-trained male volunteers	4000 mg of *P. notoginseng* capsules	Lower IL-6 concentrations 24 h after the downhill run in the placebo group.

Matsumura et al., 2015 [34]	*Zingiber officinale* Roscoe	Gingerols and shogaols	20 non-weight trained participants	4 g of ginger once a day for 5 days	Acceleration in the recovery of muscle strength following intense exercise.

**Table 5:** Botanicals with antifatigue activity on skeletal muscle.

Paper	Botanical	Compound	Model	Physiology	Main results
In vitro					

Jung et al., 2012 [38]	Korean mistletoe (*Viscum album* subsp. *coloratum*)	KME (Korean mistletoe extract)	L6 cells and C2C12 cells, mice	6 *µ*g/mL	Acceleration of OCR (37%). Significant increase in PGC-1*α* mRNA expression. 9.3-fold increase in SIRT1 expression.

Nallamuthu et al., 2014 [39]	*Aegle marmelos* (L.) Corr.	Polyphenols	BALB/c mice	100, 200, and 400 mg/kg BW for 21 d	Increase in the duration of swimming time to exhaustion by 23.4 and 47.5% for medium and higher doses, respectively.

Animals					

Wang et al., 2012 [32]	Pumpkin (*Cucurbita moschata*) fruit	*C. moschata* fruit extract (CME)	Male ICR mice	0, 50, 100, and 250 mg/kg/day for 14 days	Dose-dependent increase in swimming time (*p* = 0.0006).

Tan et al., 2013 [41]	*Panax ginseng*	Ginsenoside Rb1 (GRb1)	Rat	10 mg/kg	Significant decrease of maximum grip strength of the MG group and the GG group (*p* < 0.05).

Li et al., 2013 [40]	Tao-Hong-Si-Wu-Tang (THSWT)		32 male mice	5, 10, and 20 mL/kg body weight for 28 days	Significant increase of exhaustive swimming times (*p* < 0.05).

JiPing, 2011 [37]	*Salvia officinalis*		50 rats	100, 200, and 300 mg/kg BW	Reduction of lipid peroxidation, LDH, and CK.

Yeh et al., 2014 [33]	*Angelica sinensis*	Ferulic acid	Male ICR strain mice	0.41 g/kg/day (Ex-AS1) and 2.05 g/kg/day (Ex-AS5), 6 weeks	Slight increase of grip strength (*p* = 0.0616), at the higher AS doses, longer exercise performance (1.49, *p* = 0.0116).

Huang et al., 2011 [42]	*Acanthopanax senticosus*	Eleutheroside E, eleutheroside E_2_	Five-week-old male ICR mice	500 mg/kg and 200 mg/kg; 280 mg/kg or 70 mg/kg; 70 mg/kg or 280 mg/kg	Increase of swimming time to exhaustion at high dose (*p* < 0.01).

Chen et al., 2013 [21]	*Rubus parvifolius* L. (RPL)	Three saponins (nigaichigoside, suavissimoside, and coreanoside)	Five-week-old male	40 mg/kg and 20 mg/kg	Delays of SUN and LA accumulation, decrease in TG level, and increase in HG and LDH. Suppression of inflammatory cytokine production.

Jung et al., 2012 [38]	Korean mistletoe (*Viscum album* subsp. *coloratum*)		ICR mice	KME at 400 or 1000 mg/(kg·d) for 1 week and 25, 40, 200, and 400 mg/kg	Induction of mitochondrial activity and improvement in endurance.

Jackson et al., 2011 [51]	*Vitis vinifera*	Resveratrol	Middle-aged (18 months old) C57/BL6 mice	0.05% trans-resveratrol for 10 months	Protection against oxidative stress through the upregulation of MnSOD. Increase in the muscles activity in animals that were 28 months of age by an additional ~40% (*p* ≤ 0.05).

Humans					

Raut et al., 2012 [36]	Ashwagandha (*Withania somnifera*) (WS)		Eighteen apparently healthy volunteers	750 mg/day × 10 days; 1000 mg/day × 10 days; 1250 mg/day × 10 days	Increase in serum creatinine and blood urea nitrogen. Significant decrease in total cholesterol.

**Table 6:** Botanicals with effects on muscle atrophy.

Paper	Botanical	Compound	Model	Physiology	Main results
In vitro					

Lee et al., 2013 [45]	*Cichorium intybus* (Cii)		C2C12 myoblast	5, 10, 25, and 50 *µ*g/mL	Prevention of cell viability loss.

Hirasaka et al., 2013 [11]	*Glycine max*	Isoflavone (genistein and daidzein)	C2C12 myotubes	100 *µ*M	Approximately 2-fold increase of SIRT1 mRNA expression.

Mirza et al., 2014 [25]	*Camellia sinensis*	Epigallocatechin-3-gallate	C2C12 myotubes	10–150 *μ*M	Reduction of the expression of proteasome 19S and 20S subunits. Reduction of the expression of MuRF-1 and MAFbx.

Frati et al., 2014 [29]	Chestnuts flour	Chestnuts flour extract polyphenols or tocopherols or SL-s	C2C12 myotube cells	Polyphenols (100 nM) or tocopherols (100 nM)	Counterbalance of cell atrophy. Γ-Tocopherol and sphingolipids positively affect skeletal muscle cell atrophy.

Animals					

Vitadello et al., 2014 [43]	*Curcuma longa*	Curcumins	Wistar rats (*n *= 130)	20–40 *μ*g kg^−1^ of curcumin	About twofold increase of Grp 94 in muscles of ambulatory rats (*p* < 0.05). Counteracted loss of soleus mass and myofiber cross-sectional area by 30% (*p* ≤ 0.02).

Nakashima et al., 2014 [47]	*Chlorella*		Transgenic mice	1% *Chlorella*-supplemented diet (CSD group)	Improvement of skeletal muscle atrophy and cytochrome C oxidase activity. Recovery of body weight, enhancement of oxidative stress, and increase of CPK.

Humans					

Choquette et al., 2013 [57]	*Glycine max *	Isoflavones (daidzein, glycitein, and genistein)	70 women	Isoflavones (70 mg/day) and exercise	No effects.

**Table 7:** Botanicals with effects on muscle regeneration.

Paper	Botanical	Compound	Model	Physiology	Results
In vitro					

Hwang et al., 2015 [14]	*Broussonetia kazinoki*	Kazinol-P (KP)	C2C12 and 10T1/2 cells	KP in 2% HS for 48 h, 10–1000 nM	Increase of expression of MHC, myogenin, and Troponin-T. Increase in the level of an actively phosphorylated form of p38 MAPK (pp38) in a dose-dependent manner.

Lee et al., 2014 [15]	*Corydalis turtschaninovii*	Tetrahydropalmatine (THP)	C2C12 myoblasts and fibroblast 10T1/2	Various concentrations of THP	Enhancement of the expression of muscle-specific proteins, including MHC, MyoD, and myogenin. Increase in the levels of phosphorylated p38 MAPK.

Takeda et al., 2015 [13]	Hachimijiogan (HJG)		Murine skeletal cells	HJG treatment (1–200 *μ*g/mL)	1.23-fold increase in the cell number.

Poussard et al., 2013 [27]	*Pinus pinaster*	Natural antioxidant: short oligomers of catechin and epicatechin	Human muscle satellite cells	0.05 mg/mL	Block of the apoptosis and the protein oxidation. Recovery of HSPB1.

Animals					

Allouh, 2011 [58]	*Ferula hermonis*	Ferutinin, teferdin, teferin, and epoxy-benz	Adult male rats	60 mg/kg/rat	Significant increase in muscle weight, fiber size, and nuclear number.

Bennett et al., 2013 [52]	*Vitis vinifera*	Resveratrol (3,5,4′-trihydroxystilbene)	Thirty-six male rats	125 mg/kg/day	Favorable changes to type IIA and type IIB muscle fiber CSA and reduction of apoptotic signaling in muscles of old animal.

Alway et al., 2015 [48]	*Camellia sinensis*	Epicatechin, gallocatechin, epigallocatechin, epicatechin-3-gallate, and epigallocatechin-3-gallate	Sixty male rats	GTE (50 mg/kg body weight)	Counterbalance of the loss of hind limb plantaris muscle mass (*p* < 0.05) and tetanic force (*p* < 0.05) during HLS. Improvement of muscle fiber cross-sectional area in both plantaris (*p* < 0.05) and soleus after HLS.

Evans et al., 2010 [9]	*Camellia sinensis*	Gallocatechin, epigallocatechin, epicatechin, and epigallocatechin gallate	C57BL/6J and mdx mice	0.25% or 0.5% green tea extract	Increase in the area of normal fiber morphology (*p* ≤ 0.05). Decrease in the area of regenerating fibers (*p* ≤ 0.05).

Ballak et al., 2015 [26]	*Vitis vinifera*	Resveratrol	Mice (male C57BL/6J mice)	0.4 mg per gram body mass per day	No modification of the age-related decrease in muscle force, specific tension, or mass.

Gutierrez-Salmean et al., 2014 [53]	*Camellia sinensis*	Epicatechin	Young and old C57BL/6 male mice	1 mg/kg b.i.d.	Significant decrease of myostatin levels in young and old mice (15% and 21%, resp.). Significant decrease of SA-*β*-Gal in old SkM (22%).

Vazeille et al., 2012 [59]	*Curcuma longa*	Curcumin	Male Wistar rats	1 mg/kg body weight	Improvement of recovery during reloading.

Sung et al., 2015 [35]	*Eriobotrya japonica*	Leaf extract	Young (5-month-old) and aged (18-19-month-old) rats	50 mg/kg/day	Enhancement in MyoD, myogenin, and MyHC expression. Activation of mTOR signaling pathway, which is involved in muscle protein synthesis during myogenesis.

Humans					

Terauchi et al., 2014 [60]	Grape seeds	Proanthocyanidin of grape seeds	91 women	100 or 200 mg/d proanthocyanidin	Changes in lean mass and muscle mass from baseline to 8 weeks significantly higher in treated groups.

Gutierrez-Salmean et al., 2014 [53]	*Camellia sinensis*	Epicatechin	Human subjects (*n* = 6)	25 mg of pure Epi (~1 mg/kg/day)	Increase in bilateral hand strength of ~7%. Significant increase (49.2 ± 16.6%) in the ratio of plasma follistatin/myostatin levels.

Kim et al., 2013 [24]	*Camellia sinensis*	Catechins	128 women	540 mg of catechins daily	Significant group × time interactions in TUG (*p* = 0.005), usual walking speed (*p* = 0.007), and maximum walking speed (*p* < 0.001).

Mishra and Trikamji, 2013 [5]	*Withania somnifera*	Alkaloids and steroidal lactones	35 individuals	500 mg of the whole root extract twice daily; 750 mg twice daily	Improvement of the strength and functioning of the muscle.
